# Supplementary material for: Developmental Constraints on Genome Evolution in Four Bilaterian Model Species
Source: Genome Biol Evol. 2018 Aug 20;10(9):2266–77. doi: 10.1093/gbe/evy177 (PMC6130771; doi:10.1093/gbe/evy177)
Supplement: Supplementary Data [file evy177_supp.pdf]

**Table S1**

Expression datasets used in this study; "main": dataset used for the figures in the manuscript; "supplementary": dataset used for the figures in the supplementary materials.

| Species | *C. elegans* | | *D. melanogaster* | | *D. rerio* | | *M. musculus* | |
|---|---|---|---|---|---|---|---|---|
| Dataset | main | supplementary | main | supplementary | main | supplementary | main | supplementary |
| ref | (Gerstein et al. 2010) | (Levin et al. 2016) | (Graveley et al. 2011) | (Levin et al. 2016) | (Domazet-Loso and Tautz 2010) | (Levin et al. 2016) | (Hu et al. 2017) | (Irie and Kuratani 2011) |
| datatype | RNA-seq | RNA-seq | RNA-seq | RNA-seq | Microarray | RNA-seq | RNA-seq | Microarray |
| replicates | no | no | no | no | 2 | no | >=2 | >=2 |
| stages before MZT | 3 | 2 | 0 | 0 | 4 | 3 | 1 | 0 |
| early stages | 7 | 23 | 3 | 20 | 11 | 13 | 4 | 1 |
| middle stages | 3 | 12 | 3 | 20 | 21 | 55 | 6 | 4 |
| embryo late stages | 7 | 49 | 6 | 51 | 3 | 35 | 6 | 3 |
| post-embryo late stages | 8 | 0 | 12 | 0 | 13 | 0 | 0 | 0 |
| total stages | 28 | 86 | 24 | 91 | 52 | 106 | 17 | 8 |

# Figure S1

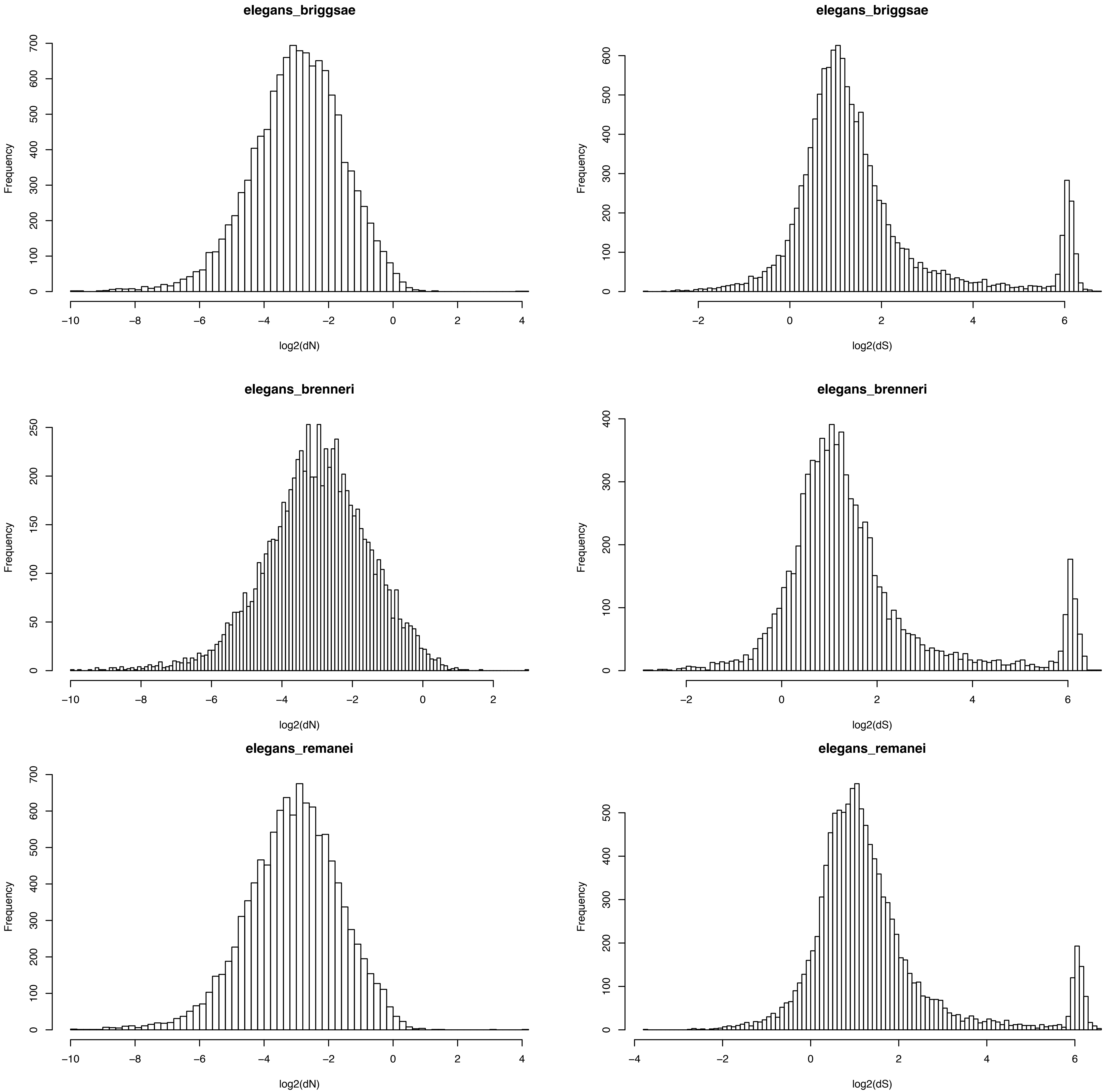
elegans_briggsae
Frequency
log2(dN)
elegans_briggsae
Frequency
log2(dS)
elegans_brenneri
Frequency
log2(dN)
elegans_brenneri
Frequency
log2(dS)
elegans_remanei
Frequency
log2(dN)
elegans_remanei
Frequency
log2(dS)

# Figure S2

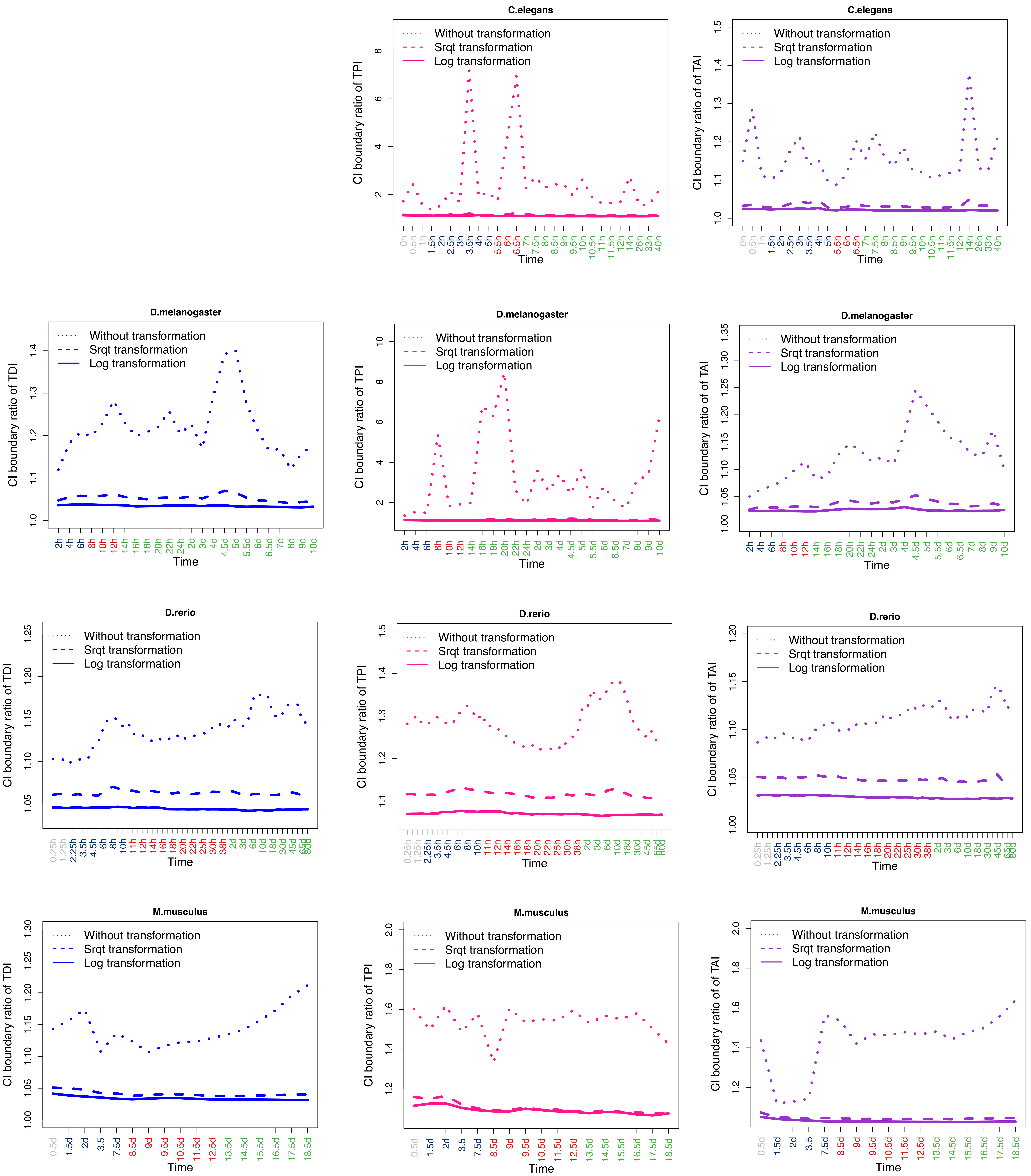

C.elegans
D.melanogaster
D.rerio
M.musculus
Without transformation
Srqt transformation
Log transformation
CI boundary ratio of TPI
CI boundary ratio of of TAI
CI boundary ratio of TDI
Time

# Figure S3

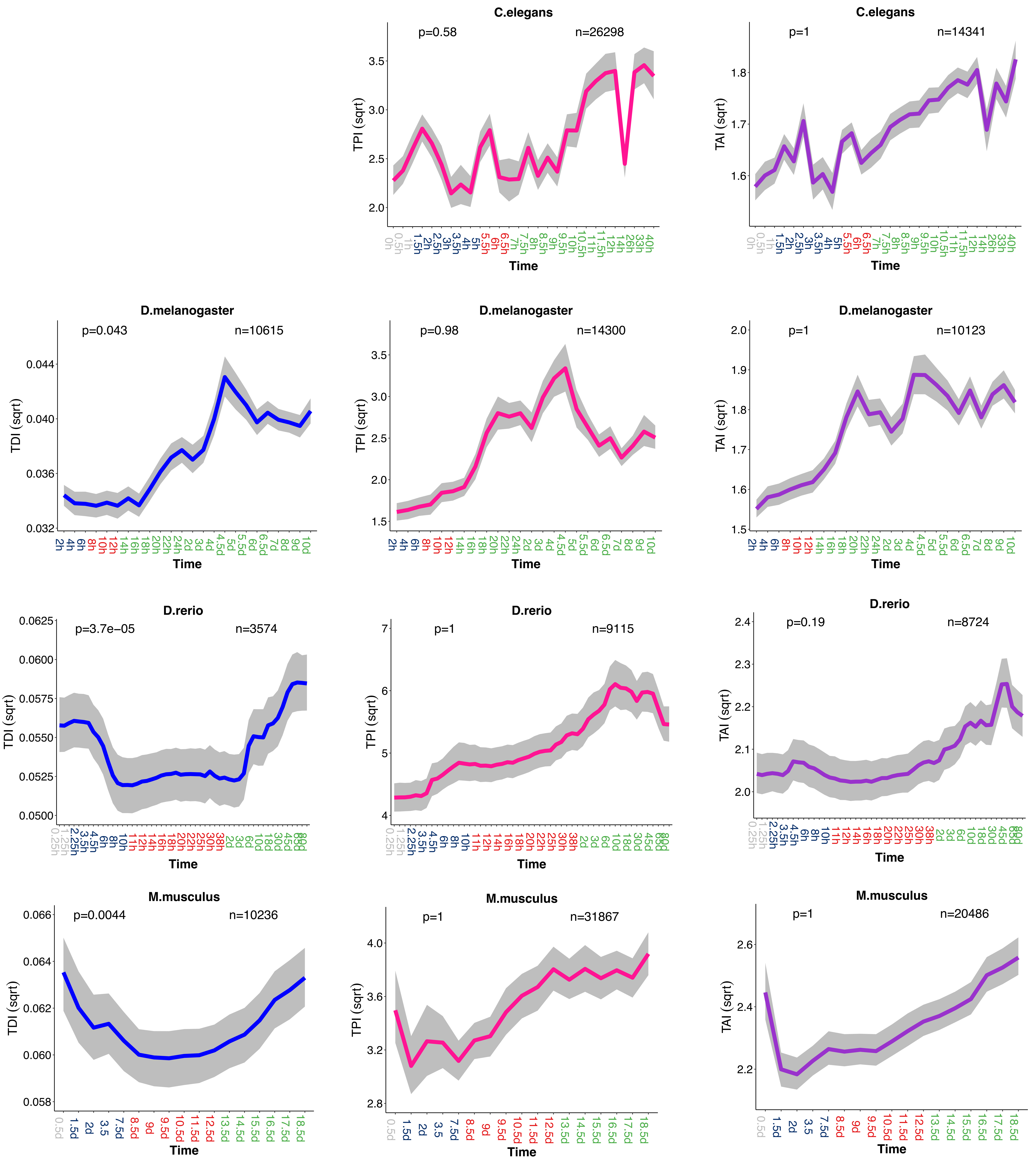
C.elegans
p=0.58
n=26298
TPI (sqrt)
Time
C.elegans
p=1
n=14341
TAI (sqrt)
Time
D.melanogaster
p=0.043
n=10615
TDI (sqrt)
Time
D.melanogaster
p=0.98
n=14300
TPI (sqrt)
Time
D.melanogaster
p=1
n=10123
TAI (sqrt)
Time
D.rerio
p=3.7e−05
n=3574
TDI (sqrt)
Time
D.rerio
p=1
n=9115
TPI (sqrt)
Time
D.rerio
p=0.19
n=8724
TAI (sqrt)
Time
M.musculus
p=0.0044
n=10236
TDI (sqrt)
Time
M.musculus
p=1
n=31867
TPI (sqrt)
Time
M.musculus
p=1
n=20486
TAI (sqrt)
Time

# Figure S4

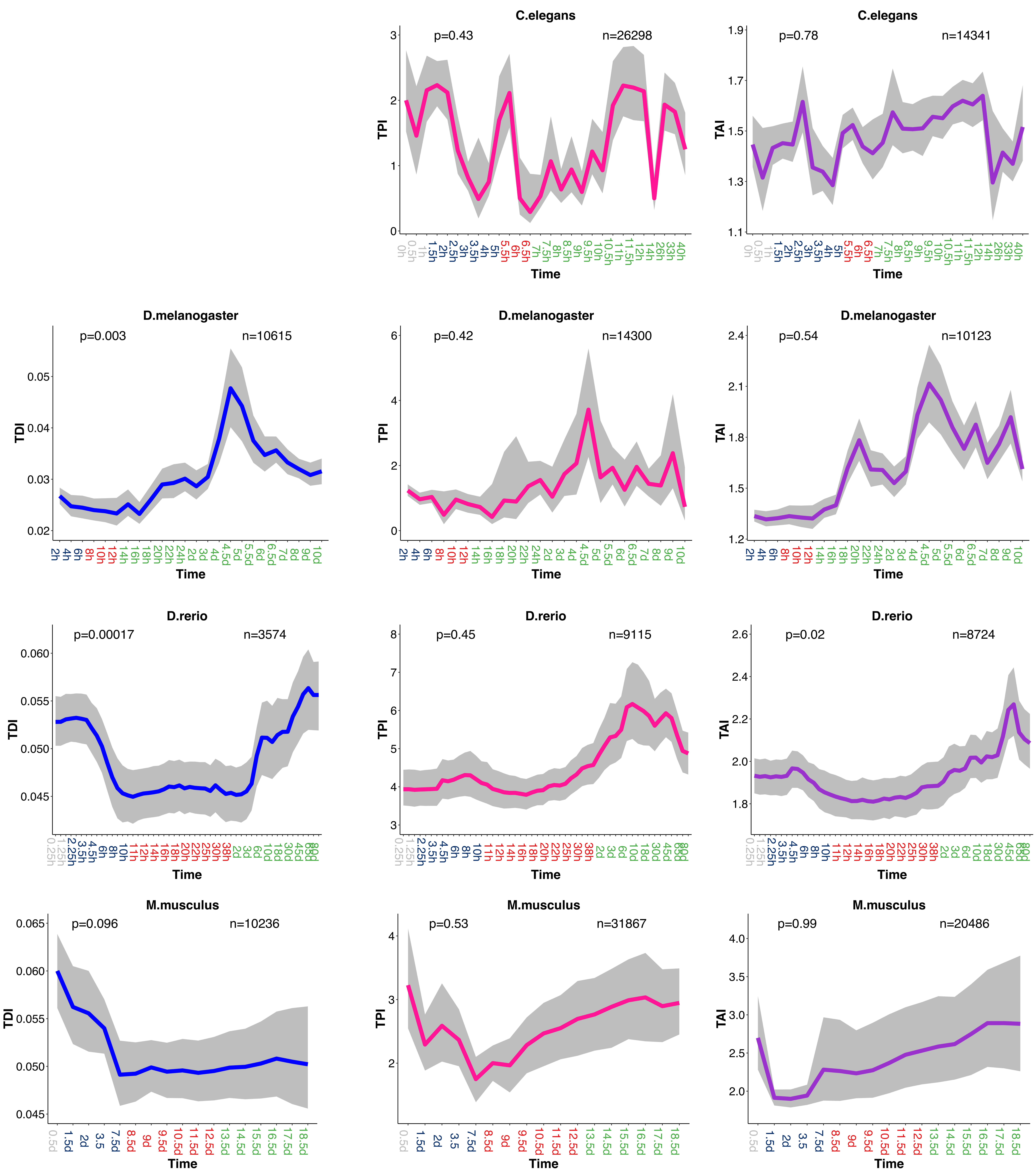

C.elegans
p=0.43
n=26298
TPI
C.elegans
p=0.78
n=14341
TAI
D.melanogaster
p=0.003
n=10615
TDI
D.melanogaster
p=0.42
n=14300
TPI
D.melanogaster
p=0.54
n=10123
TAI
D.rerio
p=0.00017
n=3574
TDI
D.rerio
p=0.45
n=9115
TPI
D.rerio
p=0.02
n=8724
TAI
M.musculus
p=0.096
n=10236
TDI
M.musculus
p=0.53
n=31867
TPI
M.musculus
p=0.99
n=20486
TAI
Time

# Figure S5

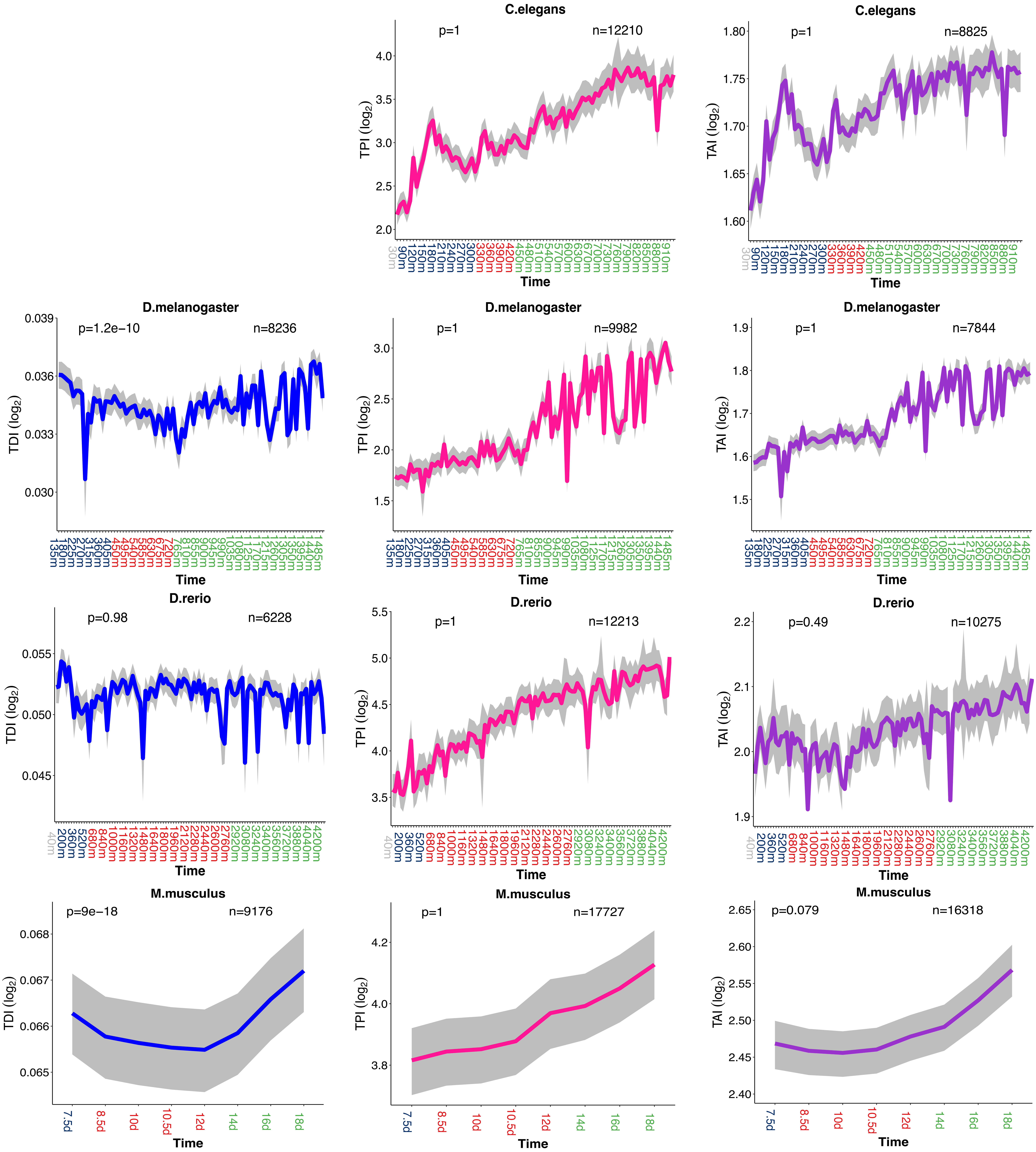

C.elegans
p=1
n=12210
TPI (log2)
Time
C.elegans
p=1
n=8825
TAI (log2)
Time
D.melanogaster
p=1.2e−10
n=8236
TDI (log2)
Time
D.melanogaster
p=1
n=9982
TPI (log2)
Time
D.melanogaster
p=1
n=7844
TAI (log2)
Time
D.rerio
p=0.98
n=6228
TDI (log2)
Time
D.rerio
p=1
n=12213
TPI (log2)
Time
D.rerio
p=0.49
n=10275
TAI (log2)
Time
M.musculus
p=9e−18
n=9176
TDI (log2)
Time
M.musculus
p=1
n=17727
TPI (log2)
Time
M.musculus
p=0.079
n=16318
TAI (log2)
Time

**Figure S6**

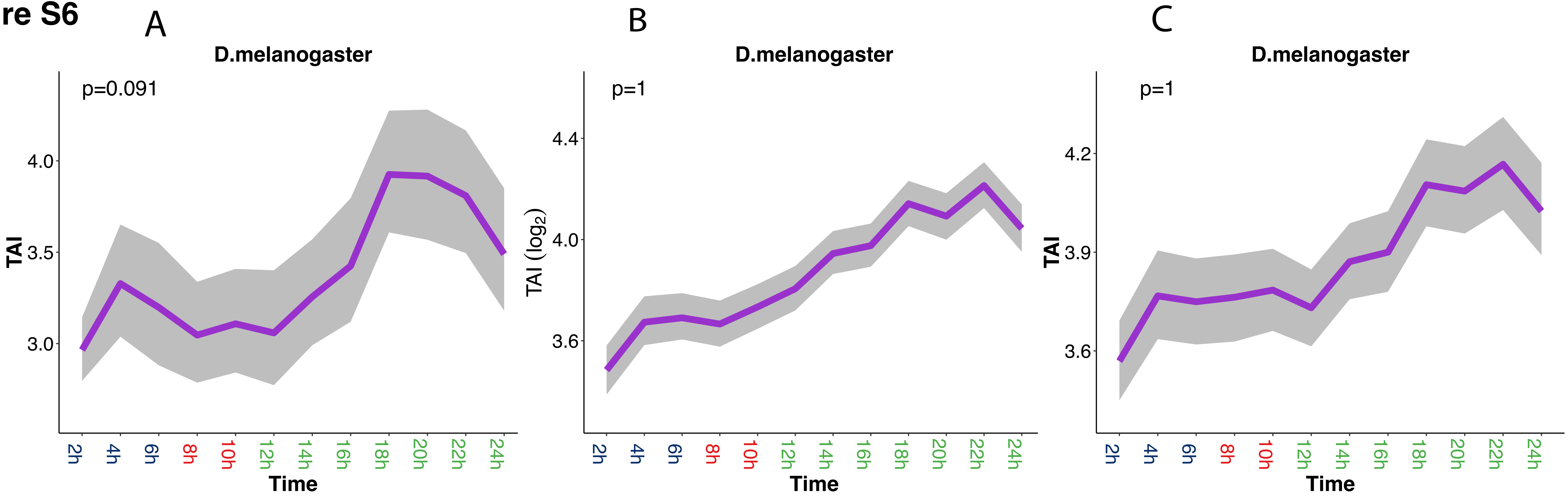

A
D.melanogaster
p=0.091
TAI
4.0
3.5
3.0
2h
4h
6h
8h
10h
12h
14h
16h
18h
20h
22h
24h
Time
B
D.melanogaster
p=1
TAI (log2)
4.4
4.0
3.6
2h
4h
6h
8h
10h
12h
14h
16h
18h
20h
22h
24h
Time
C
D.melanogaster
p=1
TAI
4.2
3.9
3.6
2h
4h
6h
8h
10h
12h
14h
16h
18h
20h
22h
24h
Time

**Figure S7**

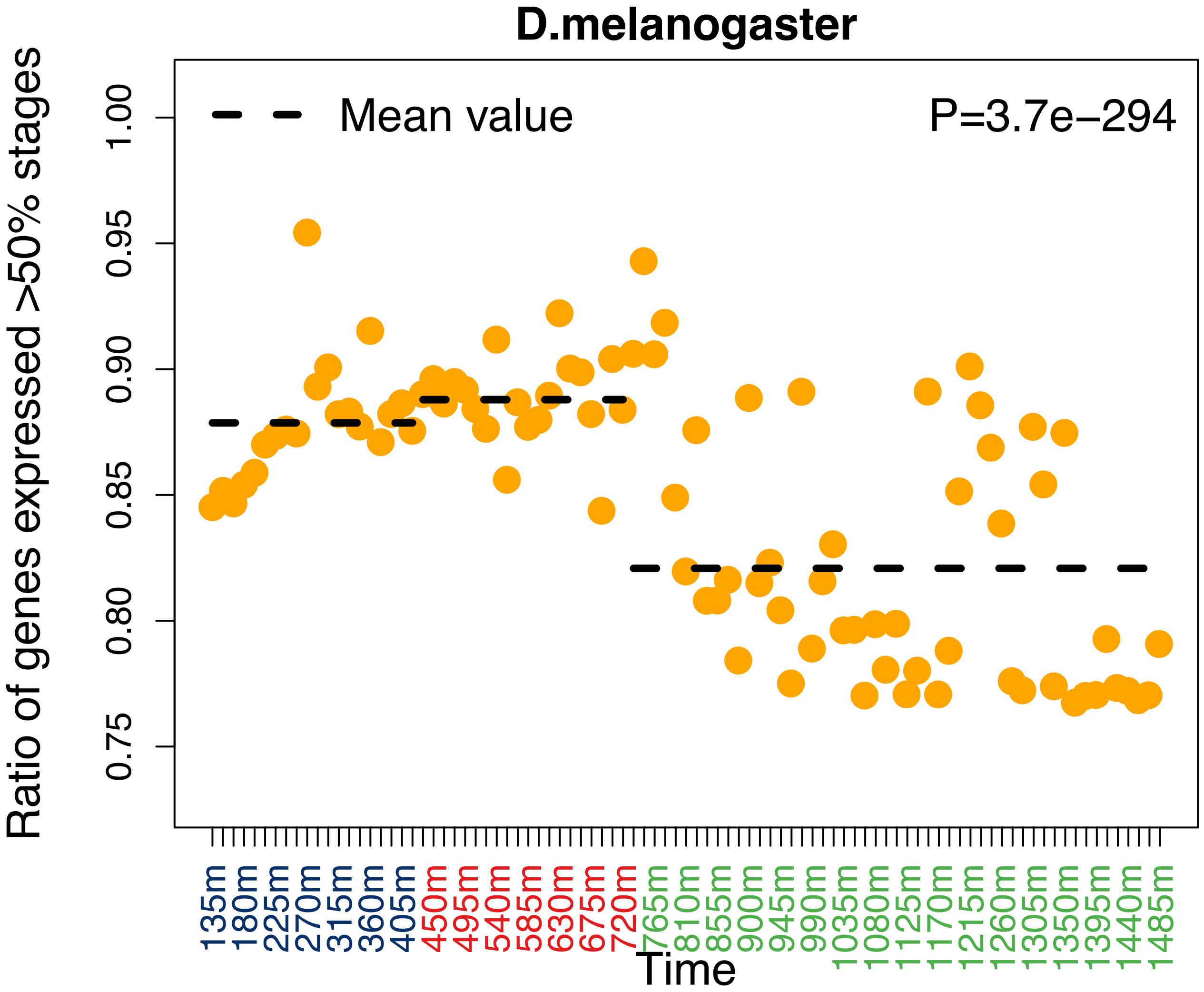
D.melanogaster
Mean value
P=3.7e−294
Ratio of genes expressed >50% stages
0.75
0.80
0.85
0.90
0.95
1.00
135m
180m
225m
270m
315m
360m
405m
450m
495m
540m
585m
630m
675m
720m
765m
810m
855m
900m
945m
990m
1035m
1080m
1125m
1170m
1215m
1260m
1305m
1350m
1395m
1440m
1485m
Time

## Figure S8

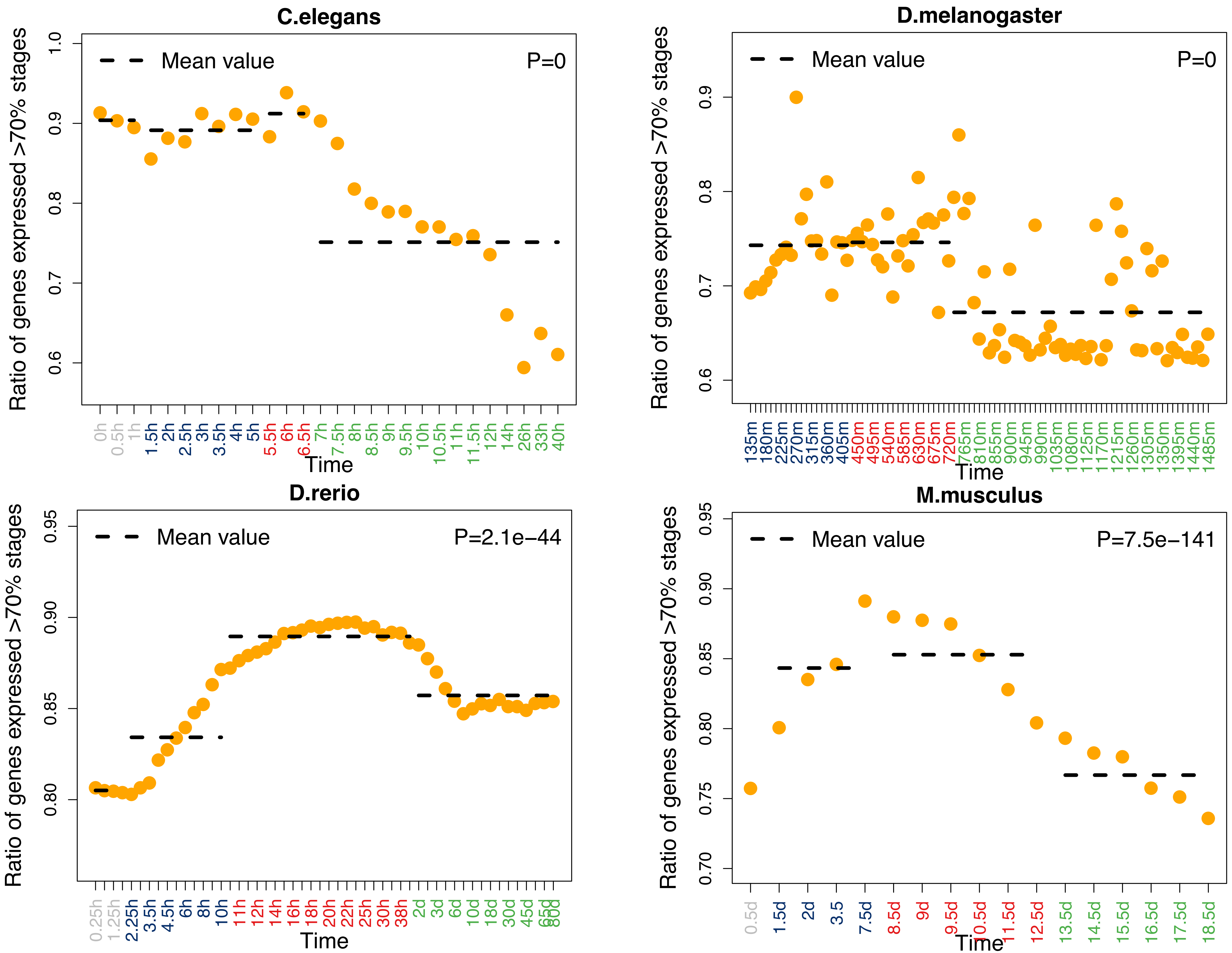
C.elegans
Mean value
P=0
Ratio of genes expressed >70% stages
Time
0h 0.5h 1h 1.5h 2h 2.5h 3h 3.5h 4h 5h 5.5h 6h 6.5h 7h 7.5h 8h 8.5h 9h 9.5h 10h 10.5h 11h 11.5h 12h 14h 26h 33h 40h
D.melanogaster
Mean value
P=0
Ratio of genes expressed >70% stages
Time
D.rerio
Mean value
P=2.1e−44
Ratio of genes expressed >70% stages
Time
M.musculus
Mean value
P=7.5e−141
Ratio of genes expressed >70% stages
Time
0.5d 1.5d 2d 3.5 7.5d 8.5d 9d 9.5d 10.5d 11.5d 12.5d 13.5d 14.5d 15.5d 16.5d 17.5d 18.5d

Figure S9

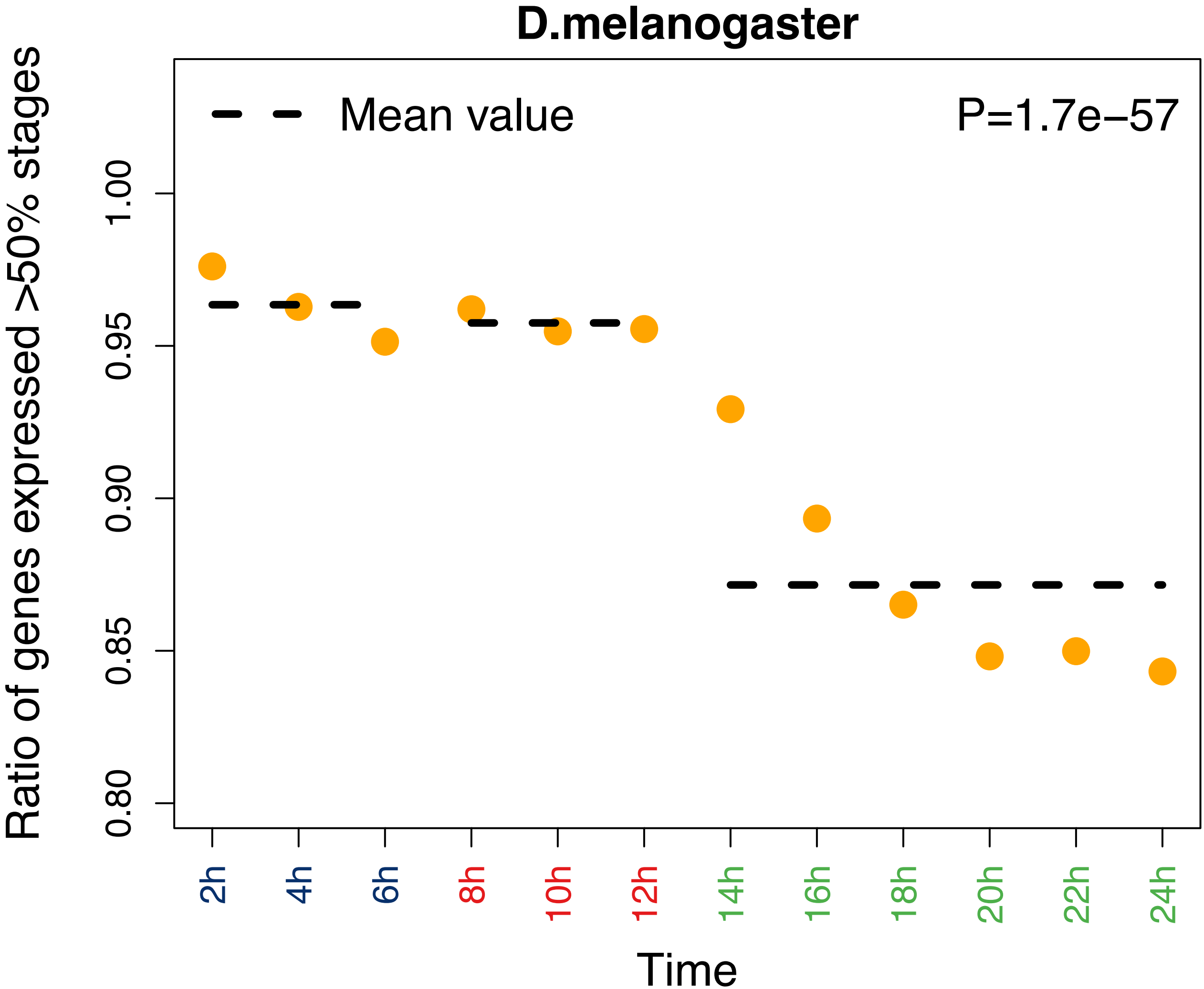
D.melanogaster
Mean value
P=1.7e−57
Ratio of genes expressed >50% stages
1.00
0.95
0.90
0.85
0.80
2h
4h
6h
8h
10h
12h
14h
16h
18h
20h
22h
24h
Time

# Figure S10

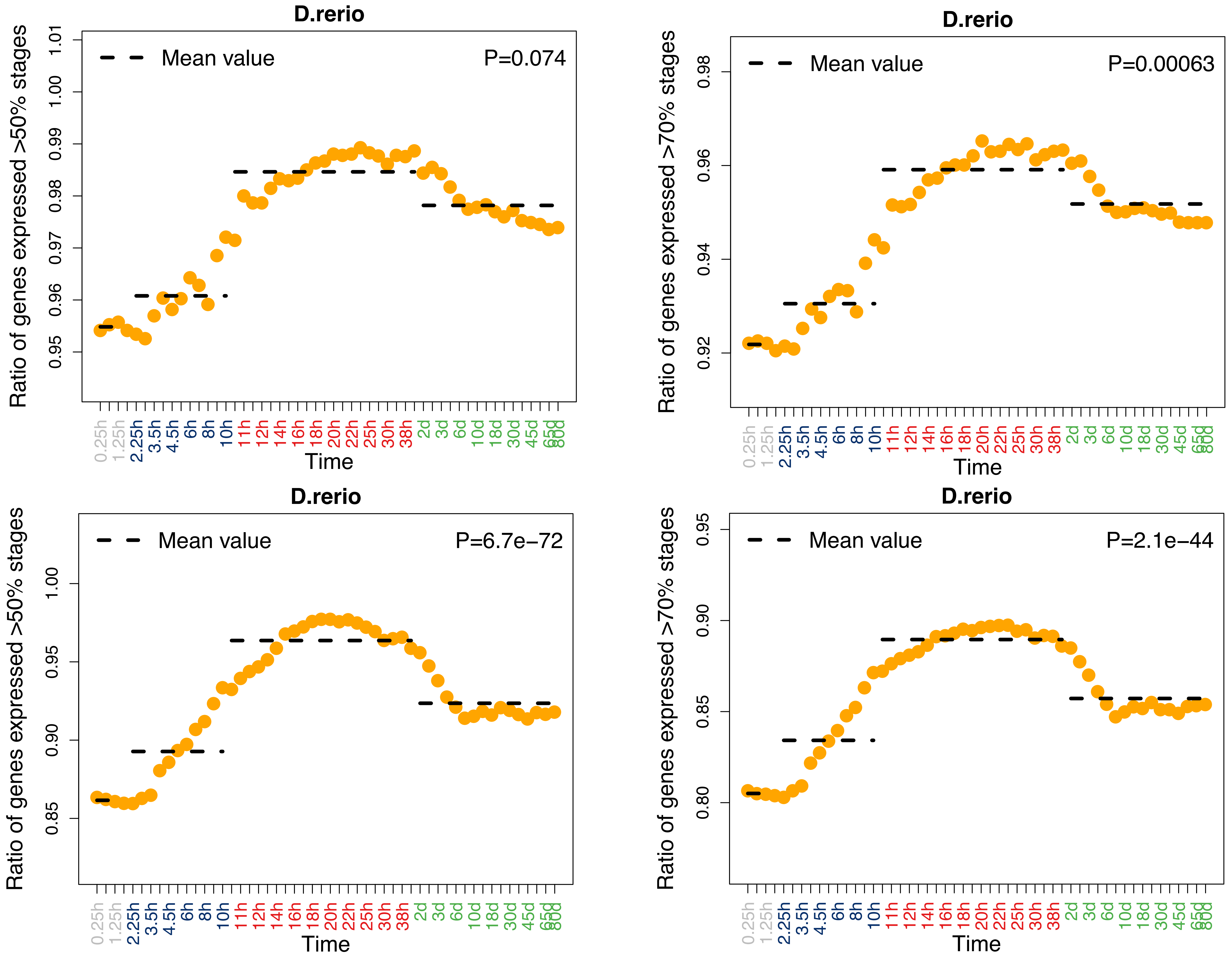
D.rerio
Mean value
P=0.074
Ratio of genes expressed >50% stages
D.rerio
Mean value
P=0.00063
Ratio of genes expressed >70% stages
D.rerio
Mean value
P=6.7e−72
Ratio of genes expressed >50% stages
D.rerio
Mean value
P=2.1e−44
Ratio of genes expressed >70% stages
Time
0.25h 1.25h 2.25h 3.5h 4.5h 6h 8h 10h 11h 12h 14h 16h 18h 20h 22h 25h 30h 38h 2d 3d 6d 10d 18d 30d 45d 65d 80d

# Figure S11

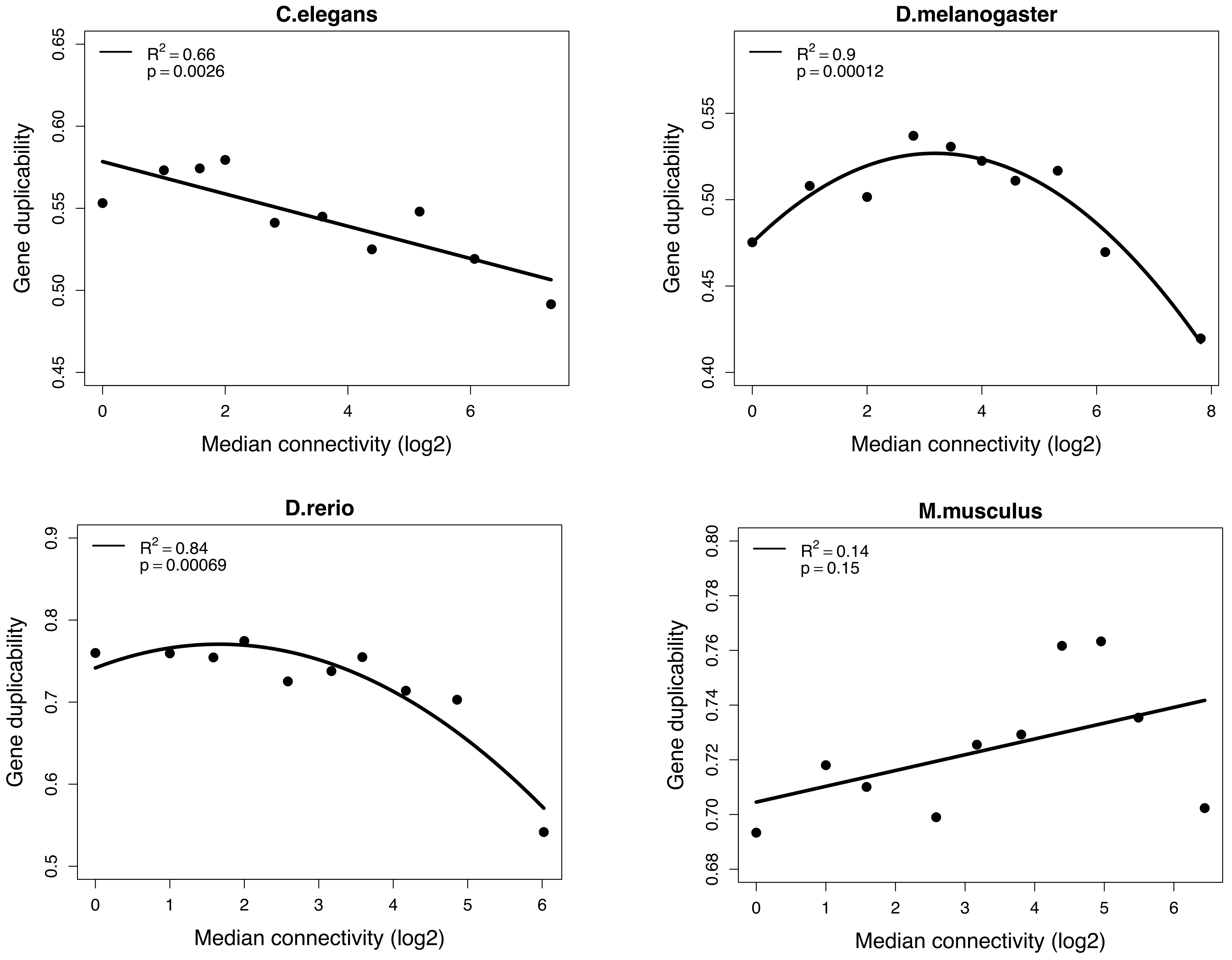

C.elegans
R2 = 0.66
p = 0.0026
Gene duplicability
Median connectivity (log2)
0.45
0.50
0.55
0.60
0.65
0
2
4
6
D.melanogaster
R2 = 0.9
p = 0.00012
Gene duplicability
Median connectivity (log2)
0.40
0.45
0.50
0.55
0
2
4
6
8
D.rerio
R2 = 0.84
p = 0.00069
Gene duplicability
Median connectivity (log2)
0.5
0.6
0.7
0.8
0.9
0
1
2
3
4
5
6
M.musculus
R2 = 0.14
p = 0.15
Gene duplicability
Median connectivity (log2)
0.68
0.70
0.72
0.74
0.76
0.78
0.80
0
1
2
3
4
5
6

**Figure S1: Distributions of dN and dS for *C. elegans*.**

**Figure S2: Comparison of 95% confidence intervals from transformed and non-transformed expression values.**

Grey, dark blue, red, and green marked time points in the x-axis represent stages before the start of MZT, early developmental stages, middle developmental stages and late developmental stages respectively. Y-axis represents the ratio of upper to lower 95% confidence interval boundary. The ratio from non-transformed expression values is plotted in dotted lines, while the ratio from $\log_2$ transformed expression values is plotted in solid lines, and the ratio from square root (abbreviated as "sqrt") transformed expression values is plotted in dashed lines.

**Figure S3: Evolutionary transcriptome indexes based on square root transformed expression values**

Legend as Figure 1, but here the indexes are based on square root transformed expression values.

**Figure S4: Evolutionary transcriptome indexes based on non-transformed expression values**

Legend as Figure 1, but here the indexes are based on non-transformed expression values.

**Figure S5: Evolutionary transcriptome indexes based on supplementary datasets.**

Legend as Figure 1, but here the indexes are based on supplementary datasets.

**Figure S6: Comparison of transcriptome phyletic age indexes (TAI).**

Dark blue, red, and green marked time points in the x-axis represent early developmental stages, middle developmental stages and late developmental stages respectively. TAI is plotted in purple line. The grey area indicates 95% confidence interval estimated from bootstrap analysis. The *p*-values for supporting the hourglass model (permutation test, early *vs*. middle development) are indicated in the top-left corner of each graph.

A: TAI based on non-transformed expression values.

B: TAI based on $log_2$ transformed expression values.

C: TAI based on non-transformed expression values, excluding the top 10% highest expressed genes.

**Figure S7: Proportion of temporal pleiotropic genes for *D. melanogaster* with the supplementary dataset.**

Legend as Figure 2, but here the result comes from the supplementary dataset of *D. melanogaster*.

**Figure S8: Proportion of temporal pleiotropic genes defined as expressed in more than 70% stages.**

Legend as Figure 2, but here the temporal pleiotropic genes are defined as expressed in more than 70% of stages.

**Figure S9: Proportion of temporal pleiotropic genes for *D. melanogaster* after removal of the second period of late development**

Legend as Figure 2.

**Figure S10: Proportion of temporal pleiotropic genes for *D. rerio* based on expressed genes defined as microarray signal rank in top 90% or 50%.**

Legend as Figure 2. In upper panel graphs, expressed genes defined as microarray signal rank in top 90%. In lower panel graphs, expressed genes defined as microarray signal rank in top 50%.

**Figure S11: Relation of protein connectivity and duplicability.**

Genes were split into 10 bins according to their connectivity. The duplicability in each bin was measured by the number of genes with paralogs divided by the number of all genes. The duplicability was fit by regression (the first degree of polynomial for *C. elegans* and *M. musculus,* while the second degree of polynomial for *D. melanogaster* and *D. rerio*), whose $R^2$ and $p$-value are indicated in the top-left corner of each graph. The median connectivity of each bin was plotted on the x-axis (in $log_2$ scale).
